# ParaCEST Agents Encapsulated in Reverse Nano-Assembled Capsules (RACs): How Slow Molecular Tumbling Can Quench CEST Contrast

**DOI:** 10.3389/fchem.2018.00096

**Published:** 2018-04-06

**Authors:** Annah Farashishiko, Jacqueline R. Slack, Mauro Botta, Mark Woods

**Affiliations:** ^1^Department of Chemistry, Portland State University, Portland, OR, United States; ^2^Dipartimento di Scienze e Innovazione Tecnologica, Università del Piemonte Orientale “Amedeo Avogadro”, Alessandria, Italy; ^3^Advanced Imaging Research Center, Oregon Health and Science University, Portland, OR, United States

**Keywords:** CEST quenching, assembled nano-capsules, NMR relaxation, molecular tumbling, contrast agents

## Abstract

Although paraCEST is a method with immense scope for generating image contrast in MRI, it suffers from the serious drawback of high detection limits. For a typical discrete paraCEST agent the detection limit is roughly an order of magnitude higher than that of a clinically used relaxation agent. One solution to this problem may be the incorporation of a large payload of paraCEST agents into a single macromolecular agent. Here we report a new synthetic method for accomplishing this goal: incorporating a large payload of the paraCEST agent DyDOTAM^3+^ into a Reverse Assembled nano-Capsule. An aggregate can be generated between this chelate and polyacrylic acid (PAA) after the addition of ethylene diamine. Subsequent addition of polyallylamine hydrochloride (PAH) followed by silica nanoparticles generated a robust encapsulating shell and afforded capsule with a mean hydrodynamic diameter of 650 ± 250 nm. Unfortunately this encapsulation did not have the effect of amplifying the CEST effect per agent, but quenched the CEST altogether. The quenching effect of encapsulation could be attributed to the effect of slowing molecular tumbling, which is inevitable when the chelate is incorporated into a nano-scale material. This increases the transverse relaxation rate of chelate protons and a theoretical examination using Solomon Bloembergen Morgan theory and the Bloch equations shows that the increase in the transverse relaxation rate constant for the amide protons, in even modestly sized nano-materials, is sufficient to significantly quench CEST.

## Introduction

In the nearly two decades since it was first demonstrated (Ward et al., 2000), exogenous Chemical Exchange Saturation Transfer (CEST) has become a topic of intense research interest. As a method of generating contrast in MRI scans, the use of exogenous CEST agents has several advantages over the more conventional relaxation agents used in clinical radiology. CEST agents can be turned on and off post-administration (Zhang et al., 2003), they can be selectively imaged even in the presence of another CEST agent (Aime et al., 2005), they can also be used to ratiometrically sense the concentration of biologically relevant species such as pH, lactate concentration, temperature or enzyme activity (Olatunde et al., 2015; Pavuluri and McMahon, 2017; Sinharay et al., 2017; Zhang et al., 2017a,b). But for all these advantages CEST still suffers from one very serious drawback: exogenous CEST agents have inherently high detection limits.

**Figure 1:**
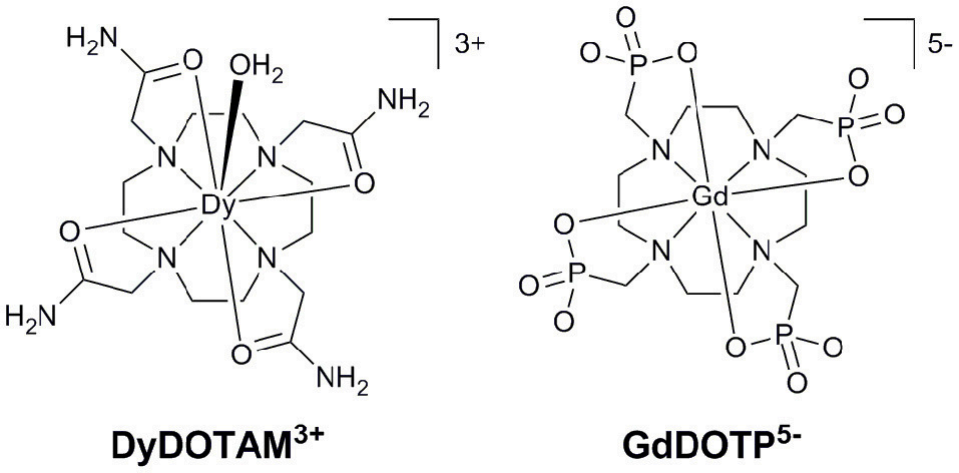
The structures of cationic **(Left)** and anionic **(Right)** Ln^3+^ chelates that have successfully been incorporated into nano-capsule delivery systems.

As its name suggests CEST occurs when a pre-saturation pulse is applied to protons that are asynchronous but exchange with the solvent water. If exchange is sufficiently slow, the saturation effect will be transferred to the solvent water, reducing its signal intensity. It is this change that is registered as increased contrast in an MRI scan. The original report on exogenous CEST examined diamagnetic agents (Ward et al., 2000). The resonance frequency of the exchanging protons on these diamagnetic agents cannot be more than a few thousand Hz at the very most from that of water and this introduces two limitations. Firstly, it limits the exchange regime; since exchange must be slow for CEST a small resonance frequency difference means that slower exchange kinetics are demanded. Intuitively it is clear that the faster the permitted exchange kinetics the more saturation will be transferred to the solvent water, increasing the amount of contrast. For this reason, larger resonance frequency differences are to be preferred. Secondly, when the resonance frequency of protons on the CEST agent is close to that of solvent water a significant amount of off-resonance direct saturation can occur. This undesirable effect interferes with the observation of CEST but can be minimized by increasing the shift separation between the two exchanging pools (Zhang et al., 2003; Woods et al., 2006). It was established shortly after the development of exogenous CEST that paramagnetic lanthanide complexes could afford CEST agents that have very large resonance frequency differences and exchange kinetics in the slow exchange regime (Zhang et al., 2001). Commonly tetraamide derivatives of DOTA, such as DOTAM (Figure 1) are used, exploiting the exchange of either the coordinated water molecule or the amide protons (Woods et al., 2006). Depending upon the particular lanthanide that is employed in the agent the shift of exchanging protons can range from a few to a few hundred ppm from water (Sherry et al., 2005). These paramagnetic CEST agents are commonly referred to as paraCEST agents.

In an effort to reduce the detection limits of exogenous CEST agents to practical levels a number of strategies have been investigated that would increase the number of exchanging protons per agent. Typically, these strategies have involved one of two approaches: either encapsulating a large volume of water within a semi-permeable membrane (such as a liposome) and using a paramagnetic shift reagent to shift the resonance frequency of the encapsulated water (Ferrauto et al., 2016); or construct a system that will carry a large payload of lower molecular weight CEST agents. In the context of paraCEST this latter strategy has the potential advantage of maintaining the large resonance frequency difference between the two exchanging proton pools. This is in contrast to lipoCEST, which has the advantage that extremely large exchanging pools can be generated. But, despite the use of a paramagnetic shift reagent, the resonance frequency difference between the two exchanging pools is quite small, comparable only to that of a diamagnetic agent (Ferrauto et al., 2017).

Reports of systems designed to carry large payloads of a low molecular weight paraCEST agent are comparatively sparse. Oligomers of Eu^3+^ based paraCEST agents have been prepared and afford the opportunity to increase the number of exchanging protons by a factor of 15–20 (Wu et al., 2008, 2010). Higher payloads have be achieved by decorating the surface of PAMAM dendrimers with paraCEST agents (Ali et al., 2009, 2012). This strategy, like that of the oligomeric agents, has two major limitations: the maximum payload of a single agent is not that high (only a few hundred at most, depending up which generation of dendrimer is used) and the chelate requires significant synthetic modifications to be undertaken before it can be incorporated into the final imaging agent. In recognition of these limitations 55 nm amine functionalized silica nano-particles (SNPs) have been used as payload delivery system (Evbuomwan et al., 2012). The amine functionalization could be used to couple directly to carboxylic acid substituents on a Eu^3+^ DOTA-tetraamide derivative. This method permitted the incorporation of a very large payload of paraCEST agents. The problem with this system was that upon incorporation into the nano-structure the CEST effect of the Eu^3+^ chelate was quenched.

We have previously demonstrated that a range of Gd^3+^ chelates with different overall negative charges can be encapsulated into different types of nano-scale capsules affording high payload delivery systems (Farashishiko et al., 2016). The benefit of this approach is that capsules are easily made from charged chelates and no expensive or laborious alterations to the chelate structure are necessary. In previous systems, a polyanionic Gd^3+^ chelate was added to a cationic polymer, such as polyallylamine hydrochloride (PAH), resulting in formation of a charge driven aggregate. A robust shell could then be added to the aggregate through either: addition of a SNPs to form Nano-Assembled Capsules (NACs) (Plush et al., 2009; Farashishiko et al., 2016); or through crosslinking with a dicarboxylic acid and peptide coupling reagent to form Cross-linked nano-Assembled Capsules (CACs) (Farashishiko et al., 2017). It was discovered that both of these approaches lead to significant improvements in the performance of the Gd^3+^ chelate as a *T*_1_-shortneing contrast agent as well as the encapsulation of a massive payload of chelate (Farashishiko et al., 2016, 2017). This observation indicates that the capsule shell in both of these systems is highly permeable to water—suggesting that either system may be amenable to modification for the delivery of high payloads of paraCEST agents.

## Experimental

### General remarks

Polyacrylic acid (PAA) (100,000 MW ~1,389 acrylic acid units per molecule, 35 wt % in water) and PAH (56,000 MW ~596 allylamine units per molecule) were purchased from Sigma-Aldrich. Ethylene diamine was purchased from J.T. Baker. A suspension of SiO_2_ NP (Snowtex-O type, 20.3 wt % SiO2 NP and pH 3.5) was purchased from Nissan chemicals and the pH adjusted to 5.5 using 150 mM NaOH prior to use. Deionized water (18.2 MΩ) was used throughout.

### Reverse NAC preparation

An aqueous solution of PAA (215 μL, 5 mg/mL) was added to an aqueous solution of DyDOTAM(Cl)_3_ (535 μL, 40 mM) in a 1.5 mL Eppendorf centrifuge tube and vortexed to mix the solutions for 10 s at low speed. This ratio of reactants afforded a charge ratio *R* = 0.5 (Equation 1). A solution of ethylene diamine (215 μL, 110 mM) was added and the mixture immediately turned turbid, indicating the formation of PAA-DyDOTAM aggregates. The reaction mixture was vortexed at a very low speed for 5 s and then aged for 10 s. An aqueous solution of PAH (20 μL, 89 μM) was added to the turbid solution and vortexed at a low speed for 10 s and aged for 15 s. An aqueous suspension of SNPs (535 μL; concentration of 2.0 wt %) was added. The resulting mixture was then stirred vigorously at a medium speed for 10 s and aged for 2 h. Finally, the RACs were purified by filter centrifugation using a 10 kDa MWCO filter centrifuge tube. The RACs so obtained were characterized in terms of size and morphology by DLS and SEM as previously described (Farashishiko et al., 2016).

### CEST experiments

CEST spectra were acquired on a Bruker Avance IIa operating at 400.13 MHz using a broadband observe (BBO) probe. Spectra were acquired with a 1 ppm spectral resolution and a relaxation delay of 10 s. Samples were prepared in a 10 M CsCl solution in H_2_O at 40 mM (Dy^3+^) and pH 6.5. The use of CsCl was to ensure that RACs did not settle during the acquisition of the CEST spectrum.

## Results and discussion

### Capsule preparation

The attraction of NACs and CACs as high payload delivery systems is the ease with which Gd^3+^ chelates are incorporated into the nano-materials. Incorporating a CEST agent in a nano-capsule presents a rather different set of challenges. It is a relatively simple matter to select a Gd^3+^ chelate as a *T*_1_-shortneing agent that can be incorporated into a nano-capsule—the only prerequisite seems to be that the overall negative change of chelate be two or greater (Farashishiko et al., 2016). The effort required to prepare a polyanionic CEST agent would be significant and would off-set the synthetic advantages of using a nano-capsule based delivery system. ParaCEST agents are commonly derived from neutral ligands, such as the tetraamide derivatives of DOTA, and are therefore intrinsically positively charged unless strategies are employed to alter the overall charge. In this context we asked the question: can the electrostatic charges that hold these nano-capsule systems be reversed to permit the delivery of a large payload of polycationic (rather than polyanionic) chelates?

**Figure 2:**
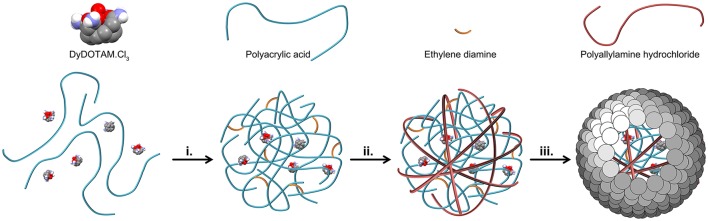
A schematic representation of the preparation of Reversed nano-Assembled Capsules (RACs). After mixing the chloride salt of DyDOTAM^3+^ with PAA, charge driven aggregation was initiated by the addition of ethylene diamine (step i.). This aggregate is then coated by addition of PAH to generate a positively charged outer surface to the aggregate (step ii.). Finally, an outer shell is added to the capsule through addition of the SNPs (step iii).

Two major hurdles blocked the seemingly simple path toward the preparation of nano-capsules loaded with a polycationic chelate. Previous results (Plush et al., 2009; Farashishiko et al., 2016, 2017) would seem to indicate that a charge driven aggregate could be produced by the simple expedient of mixing a tricationic chelate with an anionic polymer. However, when DyDOTAM^3+^ (a paraCEST agent) was added to PAA in aqueous solution at pH 7 with *R* = 0.5 the solution became only weakly turbid indicating that the aggregates that formed were only weakly charge associated. The charge ratio (*R*) is defined in Equation (1). Solution turbidity (interpreted as more robust aggregates) could be increased by addition of several equivalents of ethylene diamine to the mixture of PAA and DyDOTAM^3+^. Once aggregates have been formed in this manner, the next hurdle to overcome was developing a method for producing an encapsulating shell. Attempts to develop a cross-linked shell using EDC and polyamines such as ethylene diamine, butylene diamine and even PAH proved unsuccessful. In light of these results encapsulation strategies focused on the use of small nano-particles (Bagaria and Wong, 2011; Bagaria et al., 2011) to form a charge driven coating, *c.f*. NACs (Farashishiko et al., 2016). The use of SNPs directly with the PAA/DyDOTAM^3+^ aggregates was, unsurprisingly, not successful. Under the prevailing reaction conditions both the aggregates and the SNPs are expected to be negatively charged, and this would account for the failure of SNPs to generate stable nano-capsules in this system. To overcome this problem an intermediate step was introduced—the negatively charge aggregates were coated with a cationic polymer layer. PAH was added to the turbid mixture after formation of the PAA/DyDOTAM^3+^ aggregate. Modification of the aggregate surface charge through addition of PAH was found to facilitate formation of an encapsulating shell using SNPs. The synthetic procedure that successfully allowed the preparation of Reversed nano-Assembled Capsules (RACs) with *R* = 0.5 is described schematically in Figure 2.

(1) $$\begin{array}{r} R=\;\frac{\left|\left[chelate\right]z^{+}\right|}{\left|\left[polymer\right]z^{-}\right|} \end{array}$$

**Figure 3:**
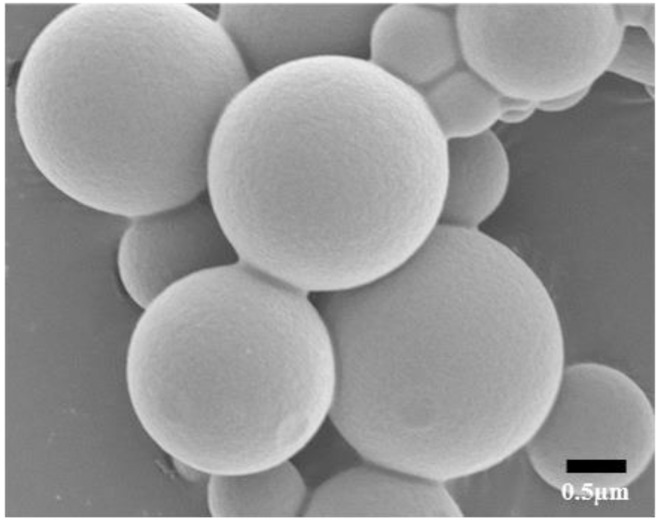
Scanning electron micrograph of DyDOTAM^3+^ containing RACs prepared at *R* = 0.5.

The capsules produced from this synthetic method were found to be quite large—the average hydrodynamic volume determined by dynamic light scattering was 650 ± 250 nm. The morphology of RACs as seen in the scanning electron micrograph (Figure 3) is very similar to that observed previously for both NACs and CACs (Farashishiko et al., 2016, 2017). It is significant to note that the rate at which water molecules were able to diffuse between the solvent and the capsule interior was found to decrease with increasing size for both NACs and CACs (Farashishiko et al., 2016, 2017). The RACs formed here are comparable in size to their NAC counterparts also formed with *R* = 0.5 (although larger than the corresponding CACs), it seems reasonable to presume that the rate at which water exchanges in and out of these capsules is comparably rapid (Farashishiko et al., 2016).

### Evaluation of CEST properties

**Figure 4:**
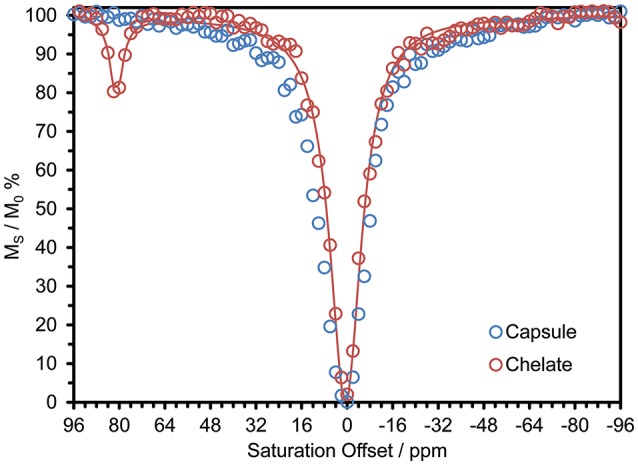
CEST spectra of 40 mM solution of DyDOTAM^3+^ at pH 6.5 and 298 K in 10 M CsCl, as discrete chelates (red, the line is a fit to the data using a 2-site exchange model of the Bloch equations Zhang et al., 2003. Fitting parameters are: τ_NH_ = 1.51 ms, *T*_1NH_ = 6 ms, *T*_1W_ = 2.3 s, *T*_2NH_ = 5 ms, *T*_2W_ = 0.31 s) and incorporated into RACs (blue) – CW irr. time = 10 s, B_0_ = 400 MHz, γB_1_ = 1000 Hz.

Previously reported systems for delivering large payloads of CEST agent (Wu et al., 2008, 2010; Ali et al., 2009, 2012; Evbuomwan et al., 2012) have tended to focus on the effect arising from exchange of water coordinated to Eu^3+^. In the context of this work the most interesting effect is that arising from exchange of the amide protons of DyDOTAM^3+^. Since DyDOTAM^3+^ has eight amide protons vs. two for a coordinated water molecule, exploiting amide based paraCEST already affords a potential four-fold increase in detectability over Eu^3+^-bound water based paraCEST. Also, because the Dy^3+^ ion induces an extremely large hyperfine shift, the resonance frequency of the amide protons of DyDOTAM^3+^ is even farther from the solvent water (~+80 ppm) than the coordinated water of Eu^3+^ (~ +50 ppm). The CEST spectrum of a 40 mM solution of DyDOTAM^3+^ at pH 6.5 demonstrates this with a clear CEST peak at about +80 ppm (Figure 4).

CEST spectra take some time to acquire and so when acquiring the CEST spectrum of nano-scale materials such as these it is necessary to ensure that the nano-material does not settle during acquisition. After experimentation it was determined that a 10 M solution of CsCl would suspend the capsules long enough to permit the acquisition of a CEST spectrum without appreciable settling of the nano-materials. The effect of even a high concentration of a chaotropic salt, such a CsCl, can be seen to be negligible from previously published data (Conte, 2015; Payne et al., 2017). Accordingly, all CEST data were recorded under identical conditions for comparative purposes. Despite these precautions, the CEST spectrum of DyDOTAM^3+^ encapsulated in RACs shows no CEST arising from the amide protons (Figure 4).

Several explanations may be offered as to why DyDOTAM^3+^, once incorporated into RACs, does not exhibit CEST arising from the amide protons. As a result of the observation that no CEST was obtained from either this nano-scale system or the Eu^3+^-loaded SNPs (Evbuomwan et al., 2012), we were motivated to probe one possible explanation in particular. Is it possible that rapid nuclear relaxation is having a quenching effect on CEST?

As the saturation pulse equalizes the populations of the α and β states, longitudinal relaxation will act to return the system to the Boltzmann distribution (Woods et al., 2006). Consequently, more rapid longitudinal relaxation will reduce the amount of CEST obtained. Although paramagnetic ions do shorten *T*_1_, the Ln^3+^ ions capable of inducing large hyperfine shifts (those of interest for paraCEST) have anisotropic *f*-shells that result in rapid electronic relaxation. Because electronic relaxation is very fast (~ 0.1–1 ps) in these ions their shortening effect on proton *T*_1_ (their relaxivity, *r*_1_) is small (Bertini et al., 1993). However, if a paramagnetic ion tumbles more slowly in solution (long rotational correlation time, τ_R_) then relaxivity can increase as a consequence of Curie nuclear spin relaxation (Banci et al., 1991). But, in the context of CEST, it is important to recognize that this effect is highly field dependent and as B_0_ increases the effect of increasing τ_R_ decreases such that, at the fields typically used for evaluating CEST agents (9.4 T and above), τ_R_ has virtually no effect on *r*_1_ (ω_H_τ_R_ >> 1). It is therefore not expected that coupling a paraCEST agent to a nano-scale system will alter proton *T*_1_s in a way that will adversely affect CEST.

**Figure 5:**
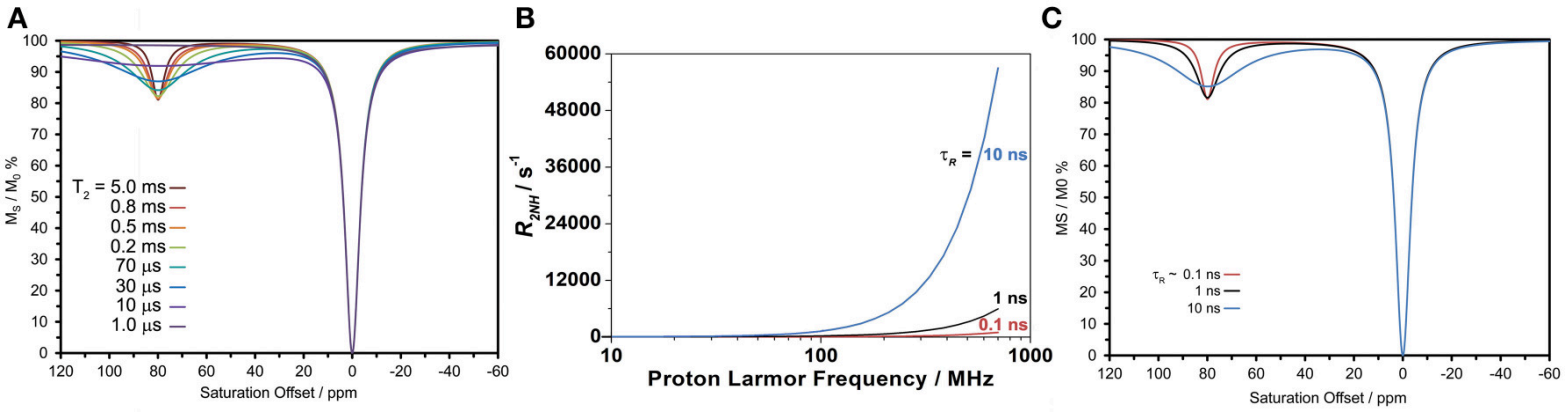
Calculated properties of DyDOTAM^3+^ under different conditions. **(A)** Simulations of CEST arising from the amide protons using the Bloch equations to assess the potential effect of shortening *T*_2NH_, generated fixing all parameters to similar values to those obtained from fitting the experimental spectrum in Figure 4 (*B*_0_ = 400 MHz, γ*B*_1_ = 1000 Hz, $T_{1}^{\text{bulk}}$ = 2 s, $T_{2}^{\text{bulk}}$ = 1 s, *T*_1NH_ = 40 ms, *k*_ex_ = 650 s^−1^). **(B)** Simulations of the amide proton transverse relaxation rate constant as a function of field for different values of τ_R_ assuming a distance of 5 Å and an electronic relaxation time of 0.1 ps. **(C)** Simulations of CEST arising from the amide protons using the Bloch equations, the same parameters used in **(A)** and *R*_2NH_ values determined for the different τ_R_ values in **(B)**.

The effect of increasing τ_R_ on transverse relaxation stands in marked contrast to that observed for longitudinal relaxation. As predicted by theory (Banci et al., 1991), the transverse relaxivity (*r*_2_) of a paramagnetic ion with a long τ_R_ does not disperse as B_0_ increases (as *r*_1_ does), but continues to increase with the square of the magnetic field. Therefore, at high fields a paramagnetic ion that tumbles slowly could have a significantly large transverse relaxivity. Simulating the CEST spectrum of DyDOTAM^3+^ using the Bloch equations and increasingly shorter and shorter *T*_2_ values for the amide protons (*T*_2NH_) shows that a paramagnetic ion with a very substantial *T*_2_-shortening effect could potentially adversely affect CEST (Figure 5A). Note that this simulation does not take into account any *T*_2_-shortening that might occur to the solvent water protons. In the present case, the effect that DyDOTAM^3+^ has on the solvent water *T*_2_ will be highly dependent upon the rate of water exchange in and out of the RAC. Given the size of the RACs, exchange of water between the interior and solvent water is likely to be rapid which means that the *T*_2_ of solvent water is also likely to undergo a shortening effect.

Is it possible that, with slowed molecular tumbling, the *T*_2NH_ DyDOTAM^3+^ could be shortened to the extent necessary to negatively impact CEST? Using the equations of paramagnetic relaxation in a form suitable for lanthanide(III) ions (Bertini et al., 2017), *T*_2NH_ was calculated for DyDOTAM^3+^ at three different τ_R_ values: 100 ps (approximately the value of a low molecular weight chelate), and 1 and 10 ns reflecting a range of values that could be taken by smaller nanoscale materials (Figure 5B). From these calculations it can be seen that *R*_2NH_ remains very low across the plotted field range when tumbling is fast (τ_R_ = 100 ps)—it never exceeds 10^3^ s^−1^. The effect of *R*_2NH_ on the CEST arising from a rapidly tumbling chelate is negligible. However, as rotation slows the effect on *R*_2NH_ is dramatic at higher fields. For the most slowly tumbling system (τ_R_ = 10 ns) *R*_2NH_ increases substantially with increasing field—at 400 MHz *R*_2NH_ is calculated to be about 2 × 10^4^ s^−1^, in good agreement with literature values (Bertini et al., 2017). Given the considerable size of the RACs it is likely that τ_R_ for the encapsulated DyDOTAM^3+^ will be longer than the 10 ns used in this calculation, which will in turn further increase the value of *R*_2NH_–conceivably by as much as an order of magnitude. Nonetheless, these calculations provide a good basis to conservatively examine the effect that such rapid transverse relaxation could have on CEST.

Figure 5C shows simulations of CEST spectra for the amide protons of DyDOTAM^3+^ at 400 MHz using the *R*_2NH_ values obtained by calculation in Figure 5B for τ_R_ values of 0.1, 1, and 10 ns. It can be seen that the simulated CEST spectrum of the freely tumbling chelate (τ_R_ = 0.1 ns) closely resembles that obtained experimentally (Figure 4). As τ_R_ increases the CEST peak at 80 ppm broadens and decreases in intensity such that when τ_R_ = 10 ns the CEST peak is already becoming broad and less distinct. As noted earlier τ_R_ = 10 ns represents a conservatively rapid rate of tumbling for RACs with a hydrodynamic volume averaging 650 nm. If τ_R_ is longer, as seems likely, then *R*_2NH_ will be larger and the detrimental effect on CEST increased. Slow molecular tumbling in a Dy^3+^ chelates could be responsible for entirely quenching CEST in systems such as these RACs.

Not only will the amount of CEST produced by these slowly tumbling systems be reduced, but it will also be increasingly difficult to extract the CEST. Because the CEST peak is now very broad, a significant bandwidth is required of the pre-saturation pulse. In practice, this means that the power of this pulse must be high. The CEST spectrum of the DyDOTAM^3+^ loaded RACs was acquired using a healthy 1 kHz of γB_1_ power, affording a reasonable pulse bandwidth. In practice a pre-saturation power equivalent to γB_1_ = 1 kHz is too high for practical MRI applications. Lower pulse powers, with their correspondingly narrower bandwidths, will be able to saturate only a small fraction of the amide proton resonance, also contributing to a quenching of CEST in these slowly tumbling systems.

## Conclusions

If the rate of proton exchange across the RAC shell was unduly slow then it would also act to quench the CEST arising from the amide protons of the encapsulated DyDOTAM^3+^. The possibility of slow water exchange kinetics can largely be ruled out by comparison with the previously reported NACs (Farashishiko et al., 2016). The encapsulating shell of both RACs and NACs are made up of electrostatically associated SNPs. This shell has a comparatively high water permeability coefficient as demonstrated by the fact that relaxivity (which demands very rapid water exchange kinetics) is not limited by water exchange until the capsules become very large, much larger than the RACs studied here. The high relaxivity of *R* = 0.5 NACs, which are similar in size to the RACs reported here, is a clear indication that that water exchange in and out of the capsule does not limit the relaxivity and is therefore rapid enough that it will not quench CEST.

It is evident from our simulations that a substantial increase in τ_R_ does have a strong negative impact on the CEST properties of a nano-scale paraCEST material based on later Ln^3+^ ions. Slow molecular tumbling can increase the rate of transverse relaxation to such an extent that it out competes CEST and effectively quenches (or reduces) the observed CEST effect. This effect will almost certainly arise when Ln^3+^ ions, in particular the heavy late lanthanides, have large magnetic moments. These ions induce large Curie relaxation contributions and thus more rapid transverse relaxation of proximate protons. In contrast, the lighter early lanthanides have smaller magnetic moments and would therefore be less likely to induce such rapid transverse relaxation even when τ_R_ becomes long (Bertini et al., 2017). This means that the effect of long τ_R_ values are unlikely to explain the CEST quenching observed by Sherry and co-workers or Pagel and co-workers for Eu^3+^-based CEST agents (Wu et al., 2008, 2010; Ali et al., 2009, 2012; Evbuomwan et al., 2012).

In summary, cationic paraCEST agents can be incorporated into nano-scale capsules through the expedient of reversing the charges. When the paraCEST agent relies upon a late Ln^3+^ ion with a large magnetic moment, the significant increase in *R*_2_ that accompanies the increase in τ_R_ results can result in quenching of CEST. It remains to be seen whether the encapsulation of paraCEST agents based on early Ln^3+^ ions, such as Pr^3+^ or Eu^3+^, would permit the development of high payload CEST systems.

## Author contributions

All authors listed have made a substantial, direct and intellectual contribution to the work, and approved it for publication.

### Conflict of interest statement

The authors declare that the research was conducted in the absence of any commercial or financial relationships that could be construed as a potential conflict of interest.
